# Breastfeeding Self-Efficacy and the Use of Prescription Medication: A Pilot Study

**DOI:** 10.1155/2012/562704

**Published:** 2011-12-15

**Authors:** Cynthia Mannion, Deborah Mansell

**Affiliations:** School of Nursing, Faculty of Nursing, University of Calgary, 2290-2500 University Drive NW, Calgary, AB, Canada T2N 1N4

## Abstract

*Objective*. To examine the association of self-efficacy, perception of milk production, and lactating women's use of medication prescribed to increase breast milk in a cohort of 18–40-year-old mothers over six months. *Methods*. Mothers (*n* = 76) attending community clinics completed the Breastfeeding Self-Efficacy Scale and the Humenick/Hill Lactation Scale, a measure of perceived milk production, three times. *Results*. Domperidone, a dopamine antagonist, was used by 28% of participants. On average, those using domperidone had lower self-efficacy scores than those not using it (*P* < 0.05) and were more likely to have used formula (Pearson chi-square test statistic  = 6.87, df = 1, *P* < 0.05). Breastfeeding self efficacy and perception of milk production were positively correlated. *Conclusion*. Breastfeeding assessment conducted prior to prescription of galactogogues is recommended for mothers and healthy term babies. Following Baby-Friendly hospital protocols and increasing self-efficacy for lactating women may be most effective in sustaining breastfeeding. Risks and benefits of various galactogogues are discussed.

## 1. Introduction

Breastfeeding is the optimal form of nutrition for term and preterm infants [1–3]. Short- and long-term benefits are associated with reduced sudden infant death syndrome; positive immunological effects; reductions in the risks of otitis media, nonspecific gastroenteritis, severe lower respiratory tract infections, atopic dermatitis, obesity, type 1 and 2 diabetes, and childhood leukemia [1, 4]. However, exclusive breastfeeding, defined by the Public Health Agency of Canada [5] as “breastfeeding with no other liquid or solid given to the infant,” is short-lived among lactating mothers everywhere. In a study comparing results from the Listening to Mothers II (LTM2; *n* = 1563) and the Maternal Experience Survey (MES; *n* = 6421) conducted in the United States 2005 and Canada 2006, respectively, rates of exclusive breastfeeding in hospital postpartum were reported as 61.2% and 75.4% [6]. At three months this rate dropped to 42.5% for the LTM2 and 51.7% for the MES. At six months both surveys reported exclusive breastfeeding rates of less than 20% [6]. Maintenance of breastfeeding seems challenging for many women. Perception of insufficient breastmilk production may contribute to cessation rates [7, 8].

Galactogogues are substances that increase milk volume by enhancing the rate of milk production and include both medications such as domperidone, metoclopramide, and herbs such as fenugreek, blessed thistle, and fennel (Tables 1 and 2). Common indications for galactogogues usually occur where lactation is nonexistent or threatened by known causes. This includes induction of lactation for adoptive mothers, relactation after weaning, maternal hypothyroidism, stimulate lactation in women with neonates in the neonatal intensive care unit, and for mothers who express milk by hand or pump [7, 9, 12, 13]. 

Anecdotally, there appears to be a trend for health care professionals to recommend pharmacological measures for mothers in the community who present with reported low milk supply issues. Reported low milk supply may be alleviated by modifying maternal self-efficacy through skill improvement and knowledge development [2]. The efficacy of galactogogues on the maintenance of breastfeeding for healthy term newborns and their mothers is unknown. Galactogogues are more commonly used for re-lactation and lactogenesis for adoptive mothers and mothers of babies in neonatal intensive care [13, 14].

High intention and initiation rates of breastfeeding, exclusive or otherwise, are rarely maintained beyond six months [15]. There are a multitude of reasons women stop breastfeeding including a lack of self-confidence in breastfeeding skills, lack of functional support, low spousal support, desire to smoke, sore nipples, postpartum depression, and maternal nutritional concerns [15–17]. Women who have experienced breast surgery, most commonly breast reduction and augmentation, may not be able to produce enough milk [18]. Maternal obesity has been implicated in delayed lactogenesis [19].

The use of formula in hospitals has been linked with low breastfeeding success rates [20]. The Baby-Friendly Hospital Initiative (BFHI) was introduced by the World Health Organization and UNICEF to increase breastfeeding rates and recommends the reduction of formula use in hospitals to promote breastfeeding [21]. In 1996, in Belarus, Kramer et al. conducted a randomized trial using the model of the Baby-Friendly Hospital initiative as an intervention and found that exclusive breastfeeding and duration increased for the first year of infants' lives given exposure to BFHI compared with standard of care received in control hospitals [22]. In 2005, it was also found that following BFHI steps, duration of breastfeeding increased. Of note, their sample consisted of women who may have been interested in exclusive breastfeeding and selected hospitals with BFHI [23]. At the same time, there was high media coverage on BFHI in Sweden where the study took place so awareness might have been heightened as breastfeeding rates in non-BFHI hospitals also rose [23]. 

The use of formula in hospital and at home has long been considered a detriment to exclusive breastfeeding and breastmilk production. However, formula is available in all hospitals as there are women who do not breastfeed. A national survey conducted in the United States reported that women choose not to breastfed because of personal preference (66.3%), they face current medical/physical problems (14.9%), feeding multiples or failed breastfeeding [24–26].

Maintenance of exclusive and partial breastfeeding is challenging for many women. Worldwide, the most common reason reported by mothers for early cessation of breastfeeding is maternal perception of insufficient milk production [8, 13, 24–32]. Insufficient milk production, often referred to as insufficient milk syndrome (IMS) was initially described by Gussler and Briesmeister in 1980 and was quickly recognized by the World Health Organization as the world's largest threat to the continuation of breastfeeding [8]. The prevalence of perceived insufficient milk production by mothers is not precisely known but has been reported between 30% and 80% [32]. This reason is associated with the highest discontinuation of breastfeeding occurring as early as 1–4 weeks postpartum [33]. Maternal perception of insufficient milk production is almost never validated by measured milk volume but is a prime influence in maternal decision making to supplement with formula, discontinue breastfeeding, or use of products that stimulate milk supply.

Galactogogues include prescription and over-the-counter (OTC) drugs, or complementary and alternative medications (herbal supplements). In the United States, it is estimated that 15% of breastfeeding women have used herbal galactogogues but the extent of galactogogue use is unknown for Canadians [34]. The use of herbal galactogogues is cause for concern because users do not confide in their health care providers and may mix prescription, OTC, and herbal medications with potential for adverse effects [35, 36].

A number of herbal supplements are purported to have galactogogue properties (Table 2). In Canada, Koren et al. of the Motherisk Program, estimate that between 7 and 55% of pregnant women use herbal supplements even though the safety and efficacy of these during pregnancy and lactation are unknown [37]. A recent American study surveyed herbal supplement use in pregnant women and reported the 14% of users did not consider herbal remedies as medications but natural and therefore benign [38, 39]. However, herbal supplements often lack standard dosing and preparation, and known composition [40]. Published research is scant supporting herbs' effectiveness in increasing milk production and more importantly their safety to mother and infant. Additionally, use of herbal medications may not be disclosed to conventional health care personnel and clients may use both prescriptions and herbal supplements courting potential adverse reactions. While there is some evidence to support the safety and efficacy of select prescription drugs, much less exists for herbal supplements, and little is known regarding drug/herbal supplements interactions [41].

Women consulting health care professionals for the perception of insufficient milk production may receive a recommendation to supplement with formula and/or to use a prescription medicine. Prescribing drugs for insufficient milk has recently gained popularity among physicians and nurse practitioners, although a lack of consensus persists regarding the efficacy of prescription galactogogues and their safety for infants [28, 30, 31]. “Some providers may inappropriately recommend galactogogues prior to emphasizing the primary means of increasing the overall rate of milk synthesis (i.e., frequent feeding and complete milk removal at regular intervals).” [7, page 42]. 

The most commonly prescribed drugs are the gastrokinetic agent, domperidone, and the antiemetic, metoclopramide. Until 2010, increased milk production was an off-label use for domperidone in Canada but the Federal Drug Administration in the United States does not recommend it due to reports of arrhythmias in users and the possibility of adverse effects for infants [8, 42]. The side effects of domperidone, a dopamine antagonist, include an increase in prolactin levels, dry mouth, abdominal cramps, and headache [4, 33, 34]. Domperidone is thought not to cross the blood-brain barrier but is excreted in breast milk in low amounts [35]. Although infant exposure to domperidone is considered insignificant, evidence is scant [36]. Other drugs that have been used include antipsychotics such as sulpiride, chlorpromazine, and hormones including human growth hormone, thyrotropin releasing hormone (TRH), and oxytocin nasal spray (Table 1). 

In preparation for submission of a national grant we conducted a pilot study in 2009 using a prospective cohort design with a convenience sample of mothers. The purpose of the pilot was to examine self-efficacy, perceived milk production, and lactating women's use of medication prescribed to increase breastmilk in a cohort of 18–40-year-old mothers over six months. The pilot allowed testing of the recruitment strategy and the demographic questionnaire. This study was approved by the Conjoint Health Research Ethics Board of the University of Calgary. Permission to access the community clinics was granted by the Director, Community Health Centres, Partnerships and Services, Alberta Health Services.

## 2. Material and Methods

A convenience sample of seventy-six mothers was recruited from parent drop-in clinics at six community health centres in Calgary, Alberta, during a three-month period. Women attended the clinics for breastfeeding support and well baby checkups. Participants were literate in English, and were breastfeeding or had attempted to breastfeed a singleton infant within the previous two months. Exclusion criteria included mothers with gestational diabetes, previous breast reductions or augmentations, illnesses such as breast cancer requiring mastectomy or extended breast lump biopsies, and those who did not have a telephone. Term, healthy babies were included in the study. Excluded from the study were babies less than 37 weeks gestation, those physically compromised, or those born with abnormalities that would affect breastfeeding such as cleft lip or palate. Participants identified by public health nurses and approached by research assistants were given a package of questionnaires assessing breastfeeding self efficacy, maternal perception of insufficient milk production, and use of galactogogues. They were surveyed again by telephone at 3 and 6 months after entry. If a woman weaned within the follow-up contact time, she was asked to complete the last set of questionnaires. 

The Breastfeeding Self-Efficacy Scale, short-form (BSES) [43], measures a mother's perceived ability to breastfeed her baby. Breastfeeding self-efficacy (BSE) is defined as a mother's confidence in her perceived ability to breastfeed the baby [44]. Decreased self-efficacy is known to be involved in cessation of breast-feeding [27, 45]. It has been shown to be associated with perceived insufficient milk production [30]. The Hill and Humenick Lactation Scale (HHLS) is a direct measure of the perception women have of their own milk production [46]. A demographic information sheet designed for this study collected data on variables known to affect breastfeeding, for example, type of delivery, family support, previous breastfeeding experience, preparation for breastfeeding, and formula use at hospital and at home. 

The BSES short form is a 14-item self-report instrument where items are preceded by the phrase “I can always” and anchored with a 5-point Likert scale where 1 = not at all confident and 5 = very confident. Items are summed to produce a score ranging from 14 to 70 with higher scores indicating higher levels of breastfeeding self-efficacy [47]. The BSES has been used extensively for a decade with a variety of populations and is widely published. The short-form scale has established validity and reliability in English and three other languages [43, 44, 47]. The Cronbach's alpha coefficient for the English short form is 0.94 [43, 47]. 

Perceived milk production was measured using the HHLS. It examines maternal commitment, satisfaction, and perceived infant satiety [46]. The HHLS is a 20-item self-report instrument where all items are anchored with a 7-point Likert scale where 1 = strongly disagree and 7 = strongly agree and can be used for subscale analysis. The three subscales show moderate to high internal consistency, Cronbach's alpha coefficients: 0.75 to 0.98 [48]. Items are summed to produce a score ranging from 20 to 140 with higher scores indicating higher levels of commitment and perceived infant satiety. It has been used with diverse populations over the last fifteen years and is widely published [49, 50]. 

Information was entered into PAWS version 17 (SPSS, Inc., Chicago, IL, USA). Descriptive statistics (means, standard deviations, frequencies, and percentages) were used to characterize the sample and describe sociodemographic characteristics. A correlation matrix was calculated to determine if any socio-demographic characteristics were significantly correlated with each of the dependent variables. Potential covariates included age, parity, education, marital status, prior experience breastfeeding, reported support for breastfeeding, use of formula in hospital, prenatal class attendance, type of delivery, level of education, support, and preparation for breastfeeding (i.e., prenatal classes). Chi square test was used to explore the relationships between categorical demographic variables. The generalized estimating equation (GEE) was used to estimate multiple predictors for BSES and HHLS. This is considered an appropriate method to identify predictors in repeated measures studies. Known predictors of breastfeeding continuation such as delivery experience, prenatal classes, access to breastfeeding information, and support for partner and family were controlled for in the model. All comparisons were calculated with statistical significance set at a *P* < 0.05.

## 3. Results

On average the participants were 30 years old (range 19–40 years), initiation of exclusive breastfeeding was reported by 57%. At Time 1, entry into the study, 83% reported breastfeeding and formula feeding and their babies were between 1 and 20 weeks old. Seventeen per cent were exclusively breastfeeding. Forty-seven per cent were still breastfeeding at Time 2 but also using formula. At the end of Time 3, almost one-third of participants reported use of domperidone during breastfeeding to increase milk production (Table 3). 

Table 4 shows the parameter results for predicting BSES scores and independent variables. Women who used formula at any time had lower breastfeeding self efficacy than those who did not use formula (*P* < 0.05). Women reporting lower breastfeeding confidence used both formula and domperidone, two interventions undertaken to ensure their babies were fed but which may be reflective of the lack of confidence in the ability to exclusively breastfeed. Women who had confidence in their ability to breastfeed (high BSES scores) also had high perceived milk production scores (high HHLS scores). 

Women (*n* = 32) who used formula had lower BSES scores than those who did not (*n* = 43, *P* < 0.001, 95% CI = −11.66, −3.66). Domperidone was reported by 28% of the participants. Those who used domperidone had lower BSES scores than those who did not (*P* < 0.05, 95% CI = −10.13, −1.15). 

Women reporting lower breastfeeding confidence as measured by BSES used both formula and domperidone (Table 4). The GEE results showed that there was no significant association found between education, marital status, and formula use and HHLS scores. 

A positive association between those who used domperidone and formula was found (Pearson chi square, test statistic = 6.87, df = 1, *P* < 0.05). As years of education increased, BSES scores increased (95% CI = 0.628, 3.251, *P* < 0.05). Also, prenatal classes specific in breastfeeding information increased BSES at Time 1 only (*P* < 0.05, 95% CI = 1.455, 14.5).

## 4. Discussion

The rate of exclusive breastfeeding was low in our study—a finding similar in many other international studies. Breastfeeding combined with formula feeding was the most commonly reported method of feeding over time. Domperidone was prescribed to one third of breastfeeding women in our study. We were unaware of the particular circumstances precluding prescription but our inclusion criteria removed preterm babies, re-lactating women and those with known health situations that would have knowingly affected breastfeeding or breast milk yield. There are reports of increasing domperidone prescription for mothers of preterm babies (<31 weeks) [51], but the healthy well educated mothers in our study were from the community and their babies were term (>37 weeks) so we found the rate of prescription use in our small study high. 

We have reported a 33% rate of surgical delivery and there may be some justification in using dopamine antagonists to raise serum prolactin levels in some women who have had a cesarean section as they may lack a significant rise in prolactin levels [52]. Substances that increase prolactin levels may be effective for those women with known low serum prolactin but this test is seldom, if ever, done. There may be women who are responders and nonresponders to dopamine antagonists but this would have to be determined by challenge. 

We found that confidence in breastfeeding skills and perceived breastmilk production were positively correlated but we also report high use of domperidone. We were unable to determine if use of domperidone contributed to perceived breastmilk production. This likely had to do with our inability to access a sample of women earlier in the postpartum period and prior to use of domperidone. Women reported that prenatal classes specifically addressing breastfeeding had an important influence on self-efficacy early in the postpartum period, a finding supported earlier in a Canadian sample of breastfeeding women [53]. We support consistency of breastfeeding information offered throughout the reproductive period, beginning in prenatal classes and extending into the postpartum period or as long as the woman continues breastfeeding. 

Formula use was also associated with the use of domperidone. We found that the HHLS did not discern between combination feeding mothers and exclusively breastfeeding mother. Women can feel satisfied and confident while combining breastfeeding and formula to feed their babies; however this combination may decrease duration of breastfeeding [20].

### 4.1. Limitations

A limitation of this study is the small urban convenience sample of women from one region in Canada. Our sample recruitment was curtailed by the H1N1 pandemic at which time Canadian federal, provincial, and local health agencies recommended isolation for infants under 6 months who could not receive H1N1 immunization [54]. Women stopped attending the drop-in clinics and community clinic nurses were redeployed to H1N1 immunization clinics and our study ceased prematurely.

Nurses selecting women in the clinic may knowingly have suggested those women already using a galactagogue or those women who were motivated to participate in a breastfeeding study may have more readily self-selected to participate in this study, thereby creating selection bias. 

 In our study only one woman reported using a herb (fenugreek) to increase milk production, and this does not reflect the range of herbal use reported elsewhere [34]. We found higher education associated with higher breastfeeding self efficacy but this may be a reflection of our participants. Our study sample consisted of well-educated, socially-advantaged women with excellent medical access, attributes not shared by all breastfeeding women. Also, participants entered the study at various times postpartum and recall bias may have affected responses. The hospitals where our sample delivered were not Baby-Friendly accredited, which may have skewed the results of this pilot study as formula is readily available on the units. Mothers may have received the recommendation to supplement with formula in hospital which can decrease a mothers overall breastfeeding duration [20].

### 4.2. Implications for Practice

We recommend that for healthy term infants born to healthy mothers, prescription medication should not be a first-line response to maternal perceived insufficient milk production, a recommendation also held by the Academy of Breastfeeding Medicine [7]. The increase in prescription medications may indicate the acceptance of a “ready fix” subsequent to short assessment visits with physicians by anxious mothers [55]. Best practice indicates a physical examination, an assessment of a breastfeeding session, and an interview prior to suggesting a prescription medication to address perceived insufficient milk production [10, 55]. By following best-practice guidelines, those women who are experiencing a physiological decrease in milk production will be appropriately identified as needing the pharmacological support to maintain adequate milk production. Our results concur with others: women with increased confidence in their breastfeeding ability are more likely to persist and are less likely to question their milk production [24–26, 56]. Combating perceived insufficient milk remains an ongoing challenge for all health care professionals working with breastfeeding mothers. Improving information to postpartum mothers directly related to milk supply (fullness, timing of feeds), measures of infant satiation (satiation cues, output), and growth spurts in infants may help some mothers address breastfeeding concerns. Stressing the importance of nighttime breastfeeding in the first eight weeks postpartum, when prolactin levels are the highest may also help to decrease mothers' perception of insufficient milk [55].

## 5. Conclusion

In 2011, Protocol 9 published by the Academy of Breastfeeding Medicine Protocol Committee stated that caution should be exercised when recommending drugs to assist initiation, maintenance, or augmentation of human milk synthesis. Stronger ties in hospital to Baby-Friendly standards, increased availability of education for health care professionals, and standard practice guidelines for breastfeeding assessment prior to medication introduction into the breastfeeding dyad may promote breastfeeding self-efficacy and increased satisfaction of mothers and healthier babies.

## Figures and Tables

**Table 1:** Prescription drugs used to increase breastmilk production.

Drug (trade name)	Intended use	Mechanism	Potential side effects
Domperidone (*Motilium*)	Antiemetic treatment of reflux disease	Peripheral dopamine antagonist	(i) Maternal cardiac arrhythmia (ii) Possible neurological side effects in infants (iii) Dry mouth, abdominal cramps, and headache (iv) Not approved in the United States

Metoclopramide (*Maxeran*)	Antiemetic	Dopamine antagonist	Drowsiness, restlessness, fatigue, anxiety, insomnia, depression, sedation, and pseudo-Parkinsonism Pediatric: prolonged clearance in infants which can result in high serum levels and a risk for methemoglobinemia. Side effects are more common in children

Sulpiride (*Eglonyl*)	Schizophrenia Antipsychotic Antidepressant	Selective dopamine antagonist	Extrapyramidal reactions and sedation in adults as well as suspected potential neonatal endocrinological effects Excreted in breastmilk

Chlorpromazine (*Thorazine*)	Antipsychotic	Increases prolactin	Sedation, lethargy, and risk of apnea Pediatric: SIDS

HGH human growth hormone (Somatotropin)	Hormone purified polypeptide of recombinant DNA	Stimulates milk production	Hypoglycemia Pediatric: absorption from breastmilk is unlikely

THR thyrotrophin releasing hormone	Treatment of hypothyroidism	Affects prolactin release	Theoretically may cause hyperthyroid condition in infants

Oxytocin (*Pitocin Syntocinon*)	Endogenous nonapeptide hormone	Stimulates milk ejection reflex	Hypotension, hypertension, water intoxication and excessive uterine contractions, bradycardia, and arrhythmias Pediatric: neonatal jaundice

Metoclopramide [9], Domperidone [10], Motilium [9, 10], and Sulpiride [11].

**Table 2:** Herbs commonly associated with galactogogue properties and known interactions.

Herbals	Intended use (main effect)	Potential side effects	Potential interactions	Contraindications
Alfalfa* (*Medicago sativa) *	Tonic Rejuvenative Diuretic	Diarrhea Reversible pancytopenia Reactivates-systemic Lupus	Immune modulators	Pregnancy Allergies

Anise* (Pimpinella anisum)	Expectorant Antispasmodic Antiseptic Antiflatulence	Seizures	Anticoagulants MAO inhibitors oral contraceptives	Pregnancy: abortifacient

Black seed caraway (*Carum carvi*)	Dyspepsia, antinausea, antiflatulent incontinence galactogogue	Contact dermatitis Weak antispasmodic activity	Disulfiram	Pregnant, breastfeeding due to antispasmodic effects

Blessed thistle* (*Cnicus benedictus*)	Stimulates menstruation, antidiarrheal, antibacterial, expectorant galactogogue	Nausea, vomiting diarrhea, contact dermatitis	Antacids, H2 antagonists, proton pump inhibitors, sucralfate, insulin	Pregnant and breastfeeding

Fennel* (*Foeniculum vulgare) *	Expectorant Antispasmodic URTI	Seizures Nausea, pulmonary edema	Anticonvulsant Sun exposure	Unknown

Fenugreek* (Trigonella foenum-graecum)	GI complaints URT congestion Antidiarrheal	Uterine stimulant Hepatotoxicity Maple-syrup Urine diarrhea	Anticoagulants Antidiabetics	Pregnancy (uterine stimulant) breastfeeding

Goat's rue (Galega officinalis)	Diuretic Galactogogue Antihyperglycemic	Headache weakness nervousness	None reported	Caution for children, pregnant, and breastfeeding patients

Milk thistle* (Silybum marianum)	Dyspepsia, liver damage from chemicals	Nausea, vomiting diarrhea	Aspirin, cisplatin, disulfiram, hepatotoxic drugs	Pregnant or breastfeeding patients

Nursing Herbal Medicine Handbook, Nursing Drug Handbook Series, Springhouse Pennsylvania.

*Often herbs are used in combination, such as mother's milk tea, various combinations of fenugreek, blessed thistle, anise, coriander, fennel, marshmallow and other herbs.

**Table 3:** Sample characteristics *N = 76*.

Variable	Number of women	*n*%
Marital status		
Married	60	79
Other	16	21

Education years		
High school	14	18
Trade school	4	5
Postsecondary	55	72

Type of delivery		
Vaginal	51	67
Cesarean section	25	33

Prenatal classes		
Have you ever attended prenatal classes?	58	76
Yes		
Did you attend prenatal classes for this pregnancy?	40	53
Yes		
Did you find prenatal information useful?	67	88
Yes		

Previous breastfeeding experience		
Yes	32	42

Formula received in hospital		
Yes	32	42

When the decision to breastfeed was made?		
When I became pregnant	26	34
I was always going to breastfeed	44	58
After prenatal classes	2	3
My partner and I discussed it	4	5

Totals may not add to 100% given missing data.

**Table 4:** Parameter results for predicting BSES and independent variables.

Parameter(s) applied with BSES	95% Wald confidence interval		Hypothesis test		
	Lower	Upper	Wald chi-square	df	Sig.

Education	.628	3.251	8.399	1	0.004
Formula use	−12.801	−2.313	7.977	1	0.005
Formula use over time	.135	4.550	4.327	1	0.038
Specific prenatal breastfeeding classes	1.455	14.513	5.745	1	0.017

Level of significance *P* < 0.05.

## Abbreviations

BSE: Breastfeeding self-efficacy
BSES: Breastfeeding Self Efficacy Scale
FDA: Federal Drug Administration
HHLS: Hill and Humenick Lactation Scale
H1N1: Influenza A virus subtype
LTM2: Listening to Mothers II
MES: Maternal Experience Survey
TRH: Thyrotropin releasing hormone.
